# BibGlimpse: The case for a light-weight reprint manager in distributed literature research

**DOI:** 10.1186/1471-2105-9-406

**Published:** 2008-10-01

**Authors:** Thomas Tüchler, Golda Velez, Alexandra Graf, David P Kreil

**Affiliations:** 1 Chair of Bioinformatics, Boku University, AT-1190 Muthgasse 18, Vienna, Austria; 2 Internet WorkShop, 2921 South Cottonwood Lane, Tucson, AZ 85713, USA

## Abstract

**Background:**

While text-mining and distributed annotation systems both aim at capturing knowledge and presenting it in a standardized form, there have been few attempts to investigate potential synergies between these two fields. For instance, distributed annotation would be very well suited for providing topic focussed, expert knowledge enriched text corpora. A key limitation for this approach is the availability of literature annotation systems that can be routinely used by groups of collaborating researchers on a day to day basis, not distracting from the main focus of their work.

**Results:**

For this purpose, we have designed BibGlimpse. Features like drop-to-file, SVM based automated retrieval of PubMed bibliography for PDF reprints, and annotation support make BibGlimpse an efficient, light-weight reprint manager that facilitates distributed literature research for work groups. Building on an established open search engine, full-text search and structured queries are supported, while at the same time making shared collections of annotated reprints accessible to literature classification and text-mining tools.

**Conclusion:**

BibGlimpse offers scientists a tool that enhances their own literature management. Moreover, it may be used to create content enriched, annotated text corpora for research in text-mining.

## Background

The published biomedical literature is growing at a tremendous pace [1,2]. Although access has increased considerably with the availability of most published research in electronic form (typically in the Portable Document Format, PDF), researchers now face a considerable challenge in organizing and managing comprehensive and up to date manuscript collections.

### Existing literature management tools

Currently, a wide range of software is offered for searching and organizing published manuscripts. With approaches ranging from open-source bibliography managers for the desktop to professional online abstracting services, supported feature sets differ substantially (see Table 1).

**Table 1:** Feature comparison

	Management of personal collections	Full-text search	Bibliography-PDF match automated	Personal annotations	Shared collections	Approximate search patterns	Search with synonyms	Index browsing	Ease of I/O with external tools^10^	Free use or open-source	Requirements
BibGlimpse	Yes	Yes	Yes	Yes	Yes	Yes	No	No	Yes/Yes	Yes^11^	Bash, Perl, Apache

Abstracting services											

PubMed	Prt^1^	No	Prt^3^	No	No	No	Prt	Yes	No/Yes	Yes	Web

ISI Web of	Prt^1^	No	No	No	No	No	No	Yes	No/Yes	No	Web

Science Search engine											

Google Scholar	No	Yes^2^	Prt^3^	No	No	No	No	No	No/No	Yes	Web

Reference managers											

EndNote	Yes	No	No^4^	Yes	No	No	No	Prt	No/Yes	No	Win/Mac

RefBase	Yes	No	No	Yes	Yes	No	No	Yes^9^	No/Yes	Yes	XAMPP^12^

iPapers	Yes	Yes	No^5^	Yes	No	No	No	No	No/Yes	Yes	Mac

Social bookmarking											

CiteULike	Yes	No	No^6^	Prt^7^	Yes	No	No	Tags	No/Prt	Yes	Web

Connotea	Yes	No	No^6^	Yes	Yes	No	No	Tags	No/Prt	Yes	Web

Digital library											

Greenstone	Yes	Yes	No	Yes^8^	Yes^8^	Yes	No	Yes	Yes/Yes	Yes	Perl, Apache

Feature comparison between BibGlimpse and other bibliographic software tools. 'Prt' indicates that a feature is *partly *supported. ^1 ^Searches and selected references can be stored in collections. ^2 ^Covers the first 120 kB of open access papers and a non-disclosed list of publishers; all recent articles by Elsevier publications are, *e.g*., excluded. PubMed is indexed with a lagtime of up to a year. ^3 ^Linking bibliography to full text; not the other way around. ^4 ^For a given reference an automated online search for the corresponding full-text can be performed. ^5 ^Requires file named PMID.pdf, where PMID is the PubMed ID, to download bibliography from PubMed. ^6 ^Needs link to website, not link to PDF. Retrieval is not generic, but publisher site tailored. ^7 ^Notes are not searchable. ^8 ^Greenstone is a tool to *build *digital libraries, so library needs to be designed first. ^9 ^MySQL database can be queried directly by passing MySQL search strings. ^10 ^*Input *means that results of external tools can easily be input into the system for subsequent integrated analysis and searches. *Output *means that data in the system can be output to external tools. ^11 ^Code free for non-profit and academic use. ^12 ^Package providing PHP, MySQL and Apache for different platforms.

While Google Scholar and other web search engines provide a full-text index of public documents online [3], there is no mechanism supporting personal collections of online documents to be annotated or searched, and many articles are not even publicly available. Desktop search tools, in turn, do not facilitate the sharing of documents and their annotation between collaborators. Dedicated bibliography management software like RefBase and WikIndx (see [4]) or the popular commercial tools EndNote [5] and RefWorks [6] have a different focus: there is no support for full-text search and, depending on the tool, complex queries are not supported or data cannot be shared online. On the other hand, full-featured software for digital libraries (like Greenstone [7]) is not only difficult to set up but also impractical for casual use. Long forms to fill in, which make filing a PDF reprint considerably more tedious than just saving it to disk, reduce acceptance in day-to-day work.

With these challenges and unmet needs in mind, we here introduce the concept of a light-weight reprint manager for the joint creation and exploitation of content enriched collections of expert annotated full-text reprints. The BibGlimpse implementation provides a simple framework for distributed literature research especially designed for this purpose. In particular, besides allowing full-text searches on manuscript collections, the support for searchable personal annotations and the ability of sharing these with colleagues are key features of the system. The automated creation of bibliographic records for a simple PDF reprint, moreover, substantially facilitates the uptake of the system. Such automatic retrieval of bibliographic records from full text PDF files is a feature that has, to the best of our knowledge, not yet been implemented elsewhere.

## Implementation

**Figure 1:**
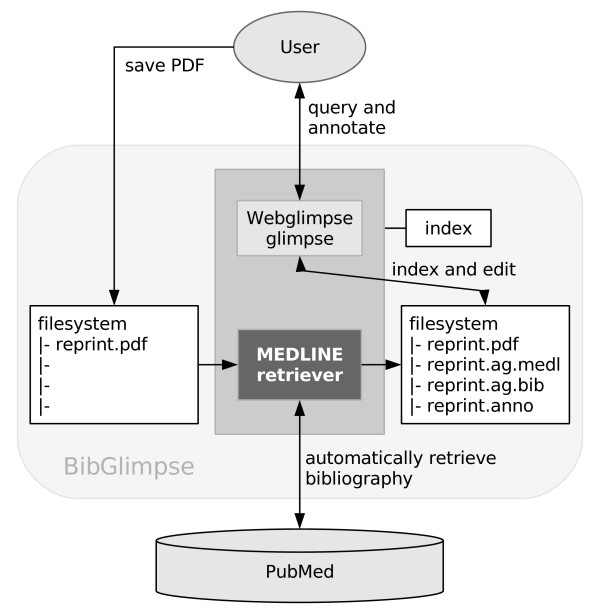
**BibGlimpse scheme**. The figure schematically illustrates how BibGlimpse incorporates automated Medline retrieval into the Webglimpse search environment. Saved PDFs are automatically matched with a Medline record and indexed. For integration with external tools, all data are directly available in flatfile format.

A defining design requirement for BibGlimpse was that users can file a new PDF reprint by *simply saving or copying it to disk*, or by uploading it online without being prompted to fill in any forms. The lightweight filesystem-based approach also means that users can easily import an entire collection of reprints using just a single command or 'drag and drop' operation for copying the directories of their PDF files. Users can of course also manually edit or add bibliographic records, personal annotation, and supplement files. Medline, BibTeX, and RIS formats are supported. When a Medline or RIS format record is available but not a BibTeX record, the system automatically creates one. Extending standard Webglimpse functionality (*cf*. Figure 1), the index is updated in the background to cover the bibliographic record, any user annotation, and the extracted full-text of an article.

### Automated Medline retrieval

When a new reprint is detected in the indexed file system, BibGlimpse extracts the plain text from the PDF file and constructs queries to automatically obtain a matching bibliographical record from PubMed. Queries are compiled with a generic pattern recognition approach, avoiding a need to prepare numerous journal specific templates for the extraction of bibliographic information from the PDF [8]. To this end, BibGlimpse first discards unspecific or irrelevant text sections, *i.e*. lines that are presumably not suited for constructing a meaningful PubMed query. This heuristic filter comprises simple rules, like a line must at least contain five characters, two words and at least one word with more than four letters. It also excludes lines that are likely to contain figure captions ('Fig.'), contact or company addresses ('Inc.'). Moreover, since citations can easily confound query construction (and are often found on the first page of articles where articles do not start on a fresh page), lines matching regular expressions that target such citations are equally removed.

By means of further heuristics, several features are then extracted from the prefiltered text in order to come up with query strings for the putative title, authors and abstract of the reprint in question: While identification of the putative manuscript title mainly focuses on the position in the text (*e.g*., the title is assumed to be located within the top lines of the document, it is supposed not to exceed a certain length and to be separated from the remainder by blank lines), features relevant for finding the manuscript's authors strongly rely on punctuation. To illustrate that, consider the following example line, obtained after converting a reprint PDF to text:

Xiaolei Yu,1 Milorad Susa,2 Cornelius Knabbe,2 Rolf D. Schmid,1 and Till T. Bachmann1*

Without knowing that 'Xiaolei' or 'Cornelius' are names, we can characterize this line by the following features: it contains 6 delimiters (including punctuation like the comma and ampersand symbols as well as the word 'and'), 5 author affiliation symbols including 1 corresponding author asterisk, 2 middle name initials (*e.g*. one in Rolf *D*. Schmid), and 10 out of 13 words start with capital letters (not counting the initials). Based on such observations, we extract the following 8 characteristic features: per-word ratios for delimiters (6/13 in this example), footnote symbols (5/13), middle name initials (2/13), and words starting with capital characters (10/13); moreover, indicators for the existence of an asterisk (1), colon characters (0), and whether the whole line is written in capital letters (0), as well as the distance to the next putative headline (counted in lines).

To construct a PubMed query string with the putative authors of a manuscript, these features are first computed for each line in the PDF and then exploited to identify 'author lines', *i.e*. lines containing the authors of a manuscript. For classification, we trained a radial basis function kernel support vector machine (SVM) on different representative training sets of 20 author and 20 other text lines. Using only the above 8 features, the finally selected support vectors achieved a respectable recall of 95% true positives (TP) with only 8% false positives (FP) as assessed in an independent test set of over 2000 candidate lines. A robust typical recall of more than 85% TP with about 10% FP in an investigation of alternative random training data indicated a well chosen feature set. Details regarding the classifier and the test corpus employed can be found in the online Supplement.

The good performance achieved in author-line identification allows targeted PubMed queries for authors with only a reasonable number of FP non-author text queries submitted. At this point, we wish to emphasize that PubMed hits are not only gathered by querying for the putative authors, but also from searching for the presumed title, abstract, and the digital object identifier, if available. The aim of the described filters is therefore not to extract the true bibliography directly from the manuscript, but rather to construct a set of query strings, that allow a retrieval of this information from PubMed with as few requests as possible.

Eventually, even obtaining a unique PubMed hit for a query string is not sufficient to assure that manuscript and bibliography match. Each retrieved Medline record is thus additionally cross-checked against the extracted full-text by reverse queries of title, authors and abstract.

Technically, the retrieval of Medline records from PubMed was implemented in Perl, such that it can be run from a single stand-alone script. This makes the feature easily accessible for other environments.

## Results and discussion

### Performance

The real-world performance of the complete system for the automated retrieval of bibliographic records was assessed on a test set of over 1000 PubMed listed manuscripts covering about 200 different journals. BibGlimpse was able to retrieve the correct PubMed records for 95% of these manuscripts with only 0.5% spurious hits and the remaining 4.5% being tagged as not-found. This shows that the combination of multiple heuristic queries and the cross-checking process yields an overall robust performance. There are some cases, however, where retrieval might not succeed. Consider the following text line returned from a 'pdftotext' conversion:

Gene Expression Profiles in Formalin-Fixed, ParaffinEmbedded Tissues Obtained with a Novel Assay for Microarray Analysis, Marina Bibikova,1 Joanne M. Yeakley,1 Eugene Chudin,1 Jing Chen,1 Eliza Wickham,1 Jessica WangRodriguez,2 and Jian-Bin g Fan1* (1 Illumina, Inc., San Diego, CA)

In this example, no newline character separates the title of the manuscript from the authors and their addresses. Moreover, the corresponding Medline entry (PMID 15563488) lists 'paraffin-embedded tissues' in the title, instead of the poorly converted 'ParaffinEmbedded' in the text version of the PDF. The cross-checking step may in such cases find no match. Also, single PDF files containing multiple short comments or letters to the editor may be assigned to the bibliography for the first comment or letter. Finally, the system may not be able to distinguish preprints from actually published manuscripts if they have the same title, authors, and abstract. In summary, however, these limitations only apply to a very small fraction of reprints files.

### Application

**Figure 2:**
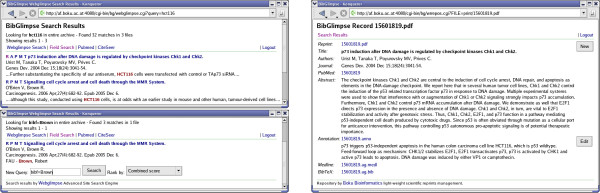
**BibGlimpse impressions**. The upper left panel shows results of a full-text query for 'HCT116'. A corresponding repository record is depicted on the right, where a domain expert captured relevant information in free-form annotation. Note that the short URLs can easily be sent to collaborating researchers. The lower-left panel demonstrates a structured query, searching only the bibliographic records for 'Brown', which avoids picking up this frequent term in the full-text, *e.g*., from the citations section.

Freeing researchers from a need to look up or enter bibliographical records and giving them an opportunity of annotating their reprints together with full-text query capabilities not only raises acceptance of the system in day-to-day usage but has profound practical implications for knowledge discovery, sharing, and retrieval. We illustrate this with a few examples (Figure 2). Important information, such as the cell line types employed, is often not contained in the abstract but can be queried by full-text search (*e.g*., a query for cell line HCT116, Figure 2). Other information may even only be *implicit *in the full manuscript text but can be captured explicitly by user annotation (*e.g*., user annotation as 'p53 wildtype').

The challenge of searching natural language text, of course, remains. Depending on the application domain, it may be valuable to consider extending the free-form annotation of articles by keywords from ontologies with controlled vocabularies or controlled subsets of natural language [9]. External tools can easily be integrated to either automatically generate these, or at least assist the user in a manual curation process [10]. This is much facilitated by having all internally stored information available as plain text files on disk. Through structured query support such additional fields can be queried directly. Having straightforward means for the integration of text-mining tools thus allows future developments to further assist users in extracting searchable knowledge from their annotated reprint collections.

The availability of installation support and minimal software prerequisites make BibGlimpse accessible for small groups of collaborating researchers. Building on Webglimpse [11], our system inherits a search engine that has been actively maintained and supported for over ten years, is easy to install and maintain using an administrative web interface, and only needs Perl, a running web server and a utility to extract plain text from PDF files, *e.g*., from 'xpdf' [12].

### Implications for text-mining

Application of BibGlimpse can make expert annotated literature collections available to text-mining. On the one hand, richly annotated text corpora are valued for training and testing algorithms [13-15]. On the other hand, text-mining applications benefit even from coarse auxiliary information [16-18]. Yet, the ability to devote scarce resources to annotating literature is often the limiting factor constraining especially machine learning approaches [19,20].

Also it is recognized that full manuscript texts provide more information than abstracts [21-24] and that information retrieval is more successful in domain specific collections [18]. Obtaining access to a comprehensive range of full-text articles, however, can be troublesome due to copyright issues, and the identification of relevant journals can be quite difficult in an interdisciplinary field [25]. Specialist researchers are actually best placed for compiling representative domain specific collections of content enriched full-text articles.

Facilitating the creation process of such annotated collections could hence significantly advance biomedical text-mining. So far, most researchers collect, freely [26] or by subscription, manuscripts of interest to their research area from multiple journals, typically by storing the PDF reprint on their computers. They maintain their personal collections of reprints, notes, and bibliographic records using a variety of tools, ranging from simple text editors or spreadsheets to commercial bibliography management software. But while extensively covering a particular area of interest, representing valuable resources of domain knowledge, such personal repositories are currently hard to search or exploit.

BibGlimpse offers researchers a tool enhancing their own routine literature management and supports the creation of shared domain-specific collections of annotated full-text manuscripts. This benefits both biological researchers as well as the text-mining community.

## Conclusion

Considering the benefits of the system's automation and query capabilities in supporting shared literature research, together with its straightforward interfacability with literature classification and text-mining tools, we have reason to expect that BibGlimpse will be widely adopted. We are confident that the concept of a light-weight reprint manager demonstrated in BibGlimpse will transform how research groups collect, manage, and share knowledge from literature research.

## Availability and requirements

Project name: BibGlimpse

Project home page: 

Operating system(s): UNIX

Programming language: Perl, Bash

Other requirements: Apache 2.2.6 or higher, Perl 5.8.6 or higher, pdftotext (*e.g*., from xpdf 3.01 or higher)

License: 

Any restrictions to use by non-academics: licence needed

## Authors' contributions

TT implemented the automated PubMed entry retrieval and drafted the manuscript, GV helped with integrating this feature into the Webglimpse search engine, AG contributed the BibGlimpse on Cygwin package, while DPK devised the concept of BibGlimpse, participated in its implementation and helped to draft the manuscript. All authors read and approved the final manuscript.
